# Physical attractiveness and sex as modulatory factors of empathic brain responses to pain

**DOI:** 10.3389/fnbeh.2015.00236

**Published:** 2015-09-07

**Authors:** Kamila Jankowiak-Siuda, Krystyna Rymarczyk, Łukasz Żurawski, Katarzyna Jednoróg, Artur Marchewka

**Affiliations:** ^1^Department of Experimental Neuropsychology, Institute of Cognitive and Behavioural Neuroscience, Faculty of Psychology, University of Social Sciences and HumanitiesWarsaw, Poland; ^2^Laboratory of Psychophysiology, Department of Neurophysiology, Nencki Institute of Experimental BiologyWarsaw, Poland; ^3^Laboratory of Brain Imaging, Neurobiology Centre, Nencki Institute of Experimental BiologyWarsaw, Poland

**Keywords:** empathy, pain, attractiveness, sex, anterior cingulate (ACC), anterior insula (AI)

## Abstract

Empathy is a process that comprises affective sharing, imagining, and understanding the emotions and mental states of others. The brain structures involved in empathy for physical pain include the anterior insula (AI), and the anterior cingulate cortex (ACC). High empathy may lead people to undertake pro-social behavior. It is important to understand how this process can be changed, and what factors these empathic responses depend on. Physical attractiveness is a major social and evolutional cue, playing a role in the formation of interpersonal evaluation. The aim of the study was to determine how attractiveness affects the level of empathy both in relation to self-rated behavior and in terms of activation of specific empathy-related brain regions. Twenty-seven subjects (14 female and 13 male) were studied using functional magnetic resonance imaging (fMRI) method while they were watching short video scenes involving physically more and less attractive men and women who exhibited pain responses. In the absence of behavioral effects in compassion ratings, we observed stronger activation in empathic brain structures (ACC; AI) for less attractive men and for attractive women than for attractive men. Evolutionary psychology studies suggest that beauty is valued more highly in females than males, which might lead observers to empathize more strongly with the attractive woman than the men. Attractive mens’ faces are typically associated with enhanced masculine facial characteristics and are considered to possess fewer desirable personality traits compared with feminized faces. This could explain why more empathy was shown to less attractive men. In conclusion, the study showed that the attractiveness and sex of a model are important modulators of empathy for pain.

## Introduction

According to the main line of psychological research, empathy is a complex process that includes affective sharing, imagining and understanding the emotions of others (Davis, 1994). Empathy may be considered a source of motivation for altruistic behavior and for cooperation (Singer and Klimecki, 2014).

An empathic response to the suffering of others can however result in two kinds of reactions: empathic distress (sometimes referred to as personal distress) and compassion (Davis, 1994; Singer and Klimecki, 2014). Empathic distress refers to personal feelings consisting of a strong aversive and self-oriented response, like anxiety and discomfort that result from observing another’s negative experience. Such a reaction could lead to protecting oneself from excessive negative feelings.

Compassion [from Latin *com* (with/together) and *pati* (to suffer)], also referred to as empathic concern, is a respondent’s feeling of warmth and concern for others. As a consequence it is associated with the motivation to help others (Singer and Klimecki, 2014). People who feel compassion in a given situation help more often than people who suffer from empathic distress (Eisenberg et al., 1989; Batson, 1991). Thus it is of great importance to understand on what factors these empathic responses depend, and to what extent, and how can they be changed.

Empathy has been studied most extensively in the domain of pain, both because of the convenience of the model, and because of the social importance attached to responding properly to the suffering of others. Research conducted on empathy for physical pain indicates the co-activation of the anterior cingulate cortex (ACC) and anterior insula (AI), which belong to the “pain matrix” (Morrison et al., 2004; Singer et al., 2004; Avenanti et al., 2005, 2006; Botvinick et al., 2005; Jackson et al., 2006; Minio-Paluello et al., 2006; Singer, 2006; Gu and Han, 2007; Lamm et al., 2007, 2011; Saarela et al., 2007; Bernhardt and Singer, 2012; Cheng et al., 2012). When taking the perspective and inferring the feelings of other people, the AI and ACC are often co-activated with the frontopolar cortex (FPC) and the dorsolateral prefrontal cortex (DLPFC; Schulte-Rüther et al., 2007; Decety and Meyer, 2008; Decety, 2010; Bernhardt and Singer, 2012; Klimecki et al., 2013), which may make it possible to predict and understand the social and affective behaviors of others (Bernhardt and Singer, 2012). Neuroimaging studies have shown that empathic brain responses vary depending on modulating factors, such as the intensity of the stimulation and the emotion displayed (de Vignemont and Singer, 2006). Stronger activations in the AI and ACC are observed when participants watch patients suffering acute compared to chronic pain (Saarela et al., 2007), and when they observe a needle deeply penetrating body parts (this stimulus is rated as high pain intensity) compared to scratching the surface of the skin (low pain intensity; Avenanti et al., 2005). However for humans, the “social animal”, what is particularly important for the extent of the empathic response is the relationship between the observed person in pain and the empathizing observer. Research conducted so far has shown that such characteristics as similarity, degree of kinship or being a member of the same or of another group, which usually leads to a greater degree of liking for others (McPherson et al., 2001), also leads to more empathy for others. Empathic neural responses increase when participants view pictures showing suffering caused by pain on the faces of people who are closely related to them (Singer and Fehr, 2005; Singer, 2006) and people they love (Cheng et al., 2010). Moreover, ACC empathic responses to perception of the pain of others are stronger when participants view the faces of racial in-group members compared with racial out-group members (Xu et al., 2009). However, there is still no data on the effect that a major social and evolutional cue such as physical attractiveness has in how strongly we empathize with other people.

Physical attractiveness plays an important role in the formation of interpersonal evaluations such as marital satisfaction and employment success (Dion et al., 1972; Berscheid and Walster, 1974). From an evolutionary perspective, attractiveness may signal fertility, health, immunity and gene quality (Buss, 1989; Shackelford and Larsen, 1999; Dixson et al., 2003; Vartanian et al., 2013). The “Beauty-is-Good” stereotype indicates that attractive people are perceived as having better personalities and being morally good, as revealed by the results of many studies (Dion et al., 1972; Eagly et al., 1991; Langlois et al., 2000; Tsukiura and Cabeza, 2011). Attractive faces seem to reward stimuli, elicit positive emotional responses, and lead to activation in such brain structures as the frontal cortex, the orbito-frontal cortex, the caudate nucleus, nucleus accumbens (NAcc) and the visual region when evaluating the attractiveness of models (Aharon et al., 2001; Kampe et al., 2001; O’Doherty et al., 2003; Kranz and Ishai, 2006; Winston et al., 2007; Vartanian et al., 2013). If this is true, one may predict that during empathizing with physically attractive people showing pain responses an activation in “pain matrix” structures and also in attractiveness evaluation structures should occur. On the other hand, pain stimuli seem to be more important evolutionally then attractiveness, and strong activation in “pain matrix” structures may well override the structures comprising the reward system. However in both scenarios it is unclear if the effect should be visible in both men and women. It is men who place a greater importance on attractiveness than women (Buss, 1989, 1994/2003; Sprecher et al., 1994). Several studies have showed sex differences in brain activation in response to attractive opposite-sex facial images contrasted with less attractive ones (O’Doherty et al., 2003; Winston et al., 2007). Only in male subjects did the medial orbitofrontal cortex (mOFC) (Cloutier et al., 2008), the ACC (Winston et al., 2007), the NAcc and the OFC (Aharon et al., 2001) show greater response to attractive faces of the opposite sex. The results suggest that it is men who find opposite-sex attractive faces more rewarding, not women (Wilson and Daly, 2004). However, meta-analysis of 32 functional magnetic resonance imaging (fMRI) studies of empathy for pain did not find any evidence for gender-specific activation differences in empathy (Lamm et al., 2011). Taking into account the abovementioned experimental findings, the question arises as to whether the degree of the brain’s empathic responses depends on both the sex and attractiveness of the model showing pain and the sex of the empathizing person.

Therefore the research project described below was aimed at determining the level of activation of specific empathy-related brain regions, how watching attractive and unattractive men and women showing pain responses affects the level of empathy.

Specifically, we tested three neurological hypotheses: (1) watching people in pain leads to activation of brain structures involved in empathy for pain; (2) attractiveness is a modulator of the empathic brain response, i.e., watching attractive people showing pain responses leads to stronger activation. Moreover, knowing that there are sex differences in brain responses to attractiveness, we expected to reveal that; (3) there are differences in the degree of activation in brain structures related to the evaluation of attractiveness in men and women when watching female and male models showing pain responses.

## Materials and Methods

### Participants

Thirty healthy right-handed subjects participated in the experiment. All participants had normal or corrected-to-normal vision and reported no history of neurological or psychiatric illnesses. Informed consent was obtained from all participants before scanning. The participants received financial compensation for participation in the study (approximately 25 euros). Three subjects were removed from the analysis due to extensive head movement, reducing the final number to 27 subjects (13 males, mean age = 25–35, SD = 3.02). The local ethics committee at the University of Social Sciences and Humanities approved this study.

### Stimuli

**Figure 1:**
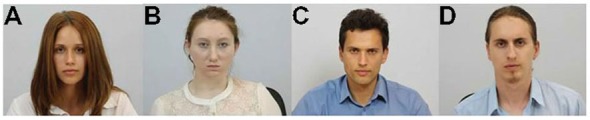
**The actors selected as (A) the more attractive woman, (B) the less attractive woman, (C) the more attractive man and (D) the less attractive man**.

To determine the effect of physical attractiveness on the level of pain empathy, a series of 6 s video clips presenting various pain-inducing situations were recorded. Each situation was endured by one of four actors who included two women (one attractive and one less attractive) and two men (one attractive and one less attractive, see Figure 1).

The process of the selection of actors in terms of attractiveness included the following steps. First, two independent judges, both of whom were psychologists, selected 15 candidates (seven women, mean age = 20–35 and eight men, mean age = 20–35) based on physical attractiveness attributes, for example facial symmetry, the shape and size of the nose and so on (Perrett et al., 1998; Rhodes, 2006; Little et al., 2011). Next, 347 subjects aged 20–40 evaluated the candidates’ attractiveness on a five-point scale that ranged from one (completely unattractive) to five (very attractive). Based on the ratings, four actors with the following average attractiveness scores were selected: attractive = 4.15 (*M* = 4.34, SD = 0.62 and *M* = 3.96, SD = 0.62, for the woman and man respectively) and non-attractive = 1.65 (*M* = 1.48, SD = 0.69 and *M* = 1.82, SD = 0.77, for the woman and man respectively). Additionally, 60 subjects between the ages of 30 and 35 evaluated the femininity and masculinity of the actors on a five-point scale that ranged from one (very low femininity/masculinity) to five (very high femininity/very masculinity). Half of the subjects rated the femininity and half rated the masculinity. Among the female actors, the femininity ratings ranged from quite low (*M* = 2.5, SD = 0.62 for the less attractive actress) to high (*M* = 4.43, SD = 0.43 for the more attractive actress), and the difference was significant (*t*_(36)_ = −15.8; *p* < 0.001). Among the male actors, the masculinity rating ranged from quite low (*M* = 2.9, SD = 0.56 for the less attractive actor) to high (*M* = 4.3, SD = 0.56 for the more attractive actor), and the difference was significant (*t*_(14)_ = 6.64; *p* < 0.001). In summary, the more attractive male model was assessed as being more masculine, and the more attractive female model was assessed as being more feminine.

The actors were filmed in four different situations (smashing their fingers, burning their lips, hitting their feet, and pricking their fingers—Pain condition). Each situation was recorded several times, and the actors were asked to present three degrees of pain intensity that included weak, moderate and most intense. After the recording sessions, the video scenes were rated by the two psychologists to select the takes that best represented the aforementioned degrees of pain intensity for each situation and for each actor. The selection of recorded stimuli was based on the three key features of facial expression, according to the Facial Action Coding System (FACS; Ekman and Friesen, 1978; Kappesser and Williams, 2002): brow lowering (AU 4); lid tighten (AU 7), and upper lip raising (AU 10) for weak and moderate pain. For intensive pain the following features were also used as additional primary criteria: cheek raising (AU 6); eyes closing (AU 43) and nose wrinkling (AU 9). In No Pain condition, the actors and the scenarios were the same, except the scenarios did not include accidents leading to pain reaction.

Finally, the film scenes were assembled such that the duration of each clip was 6 s and the duration of each element (i.e., initial presentation of the actor, the movement performed, the pain response and the facial expression of the pain felt) of each scenario was balanced.

### Experimental Paradigm

Films of the two more attractive (one female and one male) and the two less attractive (one female and one male) actors were used in the event-related fMRI paradigm. During the experiment, each actor was seen once in each of the contexts and sixteen times in total. The stimuli consisted of 64 videos (four scenes with four levels of pain intensity for the attractive and less attractive males and females). We therefore created a factorial design consisting of the following two factors: the actors’ attractiveness (two levels: more attractive vs. less attractive) and pain intensity (four levels). The order of the stimuli was quasi-randomized and then fixed for all subjects. The randomization was performed with the following constraints: the successive stimuli had to depict different situations, different pain intensity levels and different actors. Additionally, a maximum of two consecutive stimuli could depict actors of the same sex or attractiveness level.

**Figure 2:**
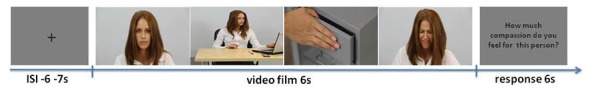
**Experimental paradigm**. Example of a single video stimulus with the more attractive female.

The stimuli were displayed using Presentation Software (Neurobehavioral Systems Inc., Albany, CA, USA) on a magnetic resonance (MR)-compatible LCD screen placed at the back of the MR scanner. The entire session lasted for approximately 19 min, and the consecutive events always followed the same structure, that is, fixation (inter-stimulus interval, ISI: 6–7 s), stimulus presentation (mean duration = 6 s) and rating of the stimulus (fixation duration = 6 s, see Figure 2 for an illustration of the scanner paradigm).

During the scanning session, the subjects were instructed to imagine the emotions of the person in the movie. After each film, a seven-point rating scale was presented and the subject was asked to rate the intensity of the compassion felt toward the person from “no compassion at all” to “very strong compassion” using two 2-button response pads (one in each hand). Operating the response pads involved moving the cursor between consecutive points at the rating scale. The rating screen was presented for 6 s. Each participant began the session after 5–10 min of training. The training consisted of learning how to operate the response pads and how to relate the response to the subject’s own feelings of compassion. At the beginning, the participant learned how to move the cursor between consecutive points on the scale, using the left and right hand alternately. Next, the participants learned how to synchronize both hands and how to accept the chosen answer. Later, they were presented with movies presenting actors and scenes differing from the experimental ones, although involving similar painful events. The participants first learned how to give the answer requested by the experimenter, and next how to give the answer corresponding to his or her natural level of compassion.

### fMRI Data Acquisition

MRI data acquisition took place at the Laboratory of Brain Imaging, Neurobiology Center, Nencki Institute of Experimental Biology on a 3-Tesla MR scanner (Siemens Magnetom Trio TIM, Erlangen, Germany) equipped with 32-channel phased array head coil. Functional data were acquired using a T2*-weighted gradient echo planar imaging (EPI) sequence with the following parameters: time repetition = 2000 ms, time echo = 30 ms, flip angle = 90°, in plane resolution = 64 × 64 mm, field of view = 224 mm, and 35 axial slices with 3.5 mm slice thickness with no gap between slices. Detailed anatomical data of the brain was acquired with a T1-weighted (T1w; time repetition = 2530 ms, time echo = 3.32 ms) sequence. Head movements were minimized with cushions placed around the participants’ heads. Three subjects were removed from the analysis due to extensive head movement (more than 7 mm—two voxels), reducing the final number to 27 subjects (13 males, mean age = 25–35, SD = 3.02).

### fMRI Data Preprocessing

Statistical Parametric Mapping (SPM12, Wellcome Trust Center for Neuroimaging, London, UK) running on MATLAB R2013b (The Math-Works Inc., Natick, MA, USA) was used for data preprocessing and the statistical analyses. First, the functional images were motion-corrected. Next, the structural images from single subjects were co-registered to the mean functional image. High-dimensional diffeomorphic anatomical registration through exponentiated lie algebra (DARTEL) was used to create a group-specific template and flow fields based on the segmented tissue from the T1w images (Ashburner, 2007). The functional images were normalized to a 2 mm isotropic voxel size using compositions of flow fields and group-specific templates (Marchewka et al., 2014). Finally, the normalized functional images were smoothed with an 8 mm isotropic Gaussian kernel. In the first-level statistical analysis, all of the experimental conditions and head movement parameters were entered into the design matrix. The data were modeled using the canonical hemodynamic response function co-involved with the experimental conditions.

Second level analyses were computed for the time window of the movie clips presentations in line with previous studies (Xu et al., 2009). All the reported data were family-wise error corrected (FWE) for multiple comparisons at the peak-level (cluster size > five voxels). Only the main peaks of activation with *T*-values within each cluster and their corresponding brain structures were reported. The numbers of voxels activated in significant clusters are presented in Table 1.

**Table 1:** **Brain regions showing activation Pain > No Pain of the Actors at *p* < 0.05 FWE corrected**.

				MNI coordinates		
	Brain region	BA	peak *T*	*x*	*y*	*z*	Cluster size
L	Thalamus	*	11.3	−15	−22	4	335
L	Lingual gyrus	BA17	10.4	−15	−91	1	301
L	Middle occipital gyrus	BA18	6.1	−21	−99	12	
L	Supplementary motor area	BA6	9.3	−6	−15	51	285
L	Anterior insula	BA13	8.4	−41	−21	18	289
L	Anterior cingulate gyrus	BA32	7.5	−5	18	45	1470
R	Supplementary motor area	BA6	7.0	8	9	63	
L	Anterior cingulate gyrus	BA32	7.0	−5	27	31	
R	Dorsolateral prefrontal cortex	BA46	7.0	48	44	16	291
R	Frontopolar cortex	BA 10	6.3	48	53	9	
R	Superior temporal gyrus	BA41	7.0	55	25	1	64
R	Declive cerebellum	*	6.8	9	−81	−6	45
L	Anterior insula	BA13	6.8	−33	23	6	308
L	Anterior insula	BA13	6.3	−41	12	−3	
R	Dorsal posterior cingulate gyrus	BA31	6.5	26	−28	39	22
L	Lentiform nucleus	Putamen	6.1	−32	−12	0	51
L	Caudate	*	6.0	−15	14	1	33
L	Brainstem	Substantia nigra	5.6	−8	−28	−17	15
L	Supramarginal-gyral	BA40	5.1	−23	−39	61	15
R	Ventral posterior cingulate gyrus	BA23	5.2	5	−22	31	6

*Abbreviation: L, left hemisphere; R, right hemisphere; BA, Brodman Area. All of the brain regions listed in this table were cluster-corrected and met the significance threshold of p < 0.05. The cluster size is the number of voxels activated in the regional cluster. Only the main peaks of activation within each cluster and their corresponding brain structures are reported*.

Additionally, the contrast estimates from the significantly activated structures in the pain > no pain contrast were extracted using the MarsBar toolbox (Brett et al., 2002). These contrast estimates were then analyzed as region of interests (ROIs) for the potential influences of the attractiveness and sex of the actors in the SPSS software.

## Results

### Behavioral Results

**Figure 3:**
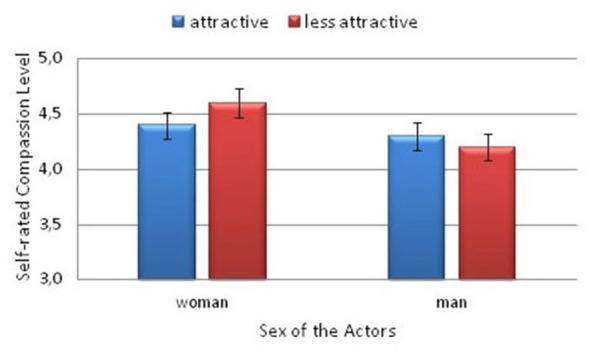
**Self-rated compassion levels felt toward the models by the participants**.

The behavioral ratings obtained during the fMRI procedure were analyzed with repeated-measures ANOVA. Evaluations of the compassion rating and reaction time (RT) means were performed with the following within-subject factors: physical trait (higher attractiveness vs. lower attractiveness) and actor sex (woman vs. man), and with the sex of the participants (woman vs. man) as a between-subject factor. In case of interaction effect the Sidak correction for multiple comparison was computed. Two-tailed *p* values are reported. The analysis of the declared compassion (empathic concern) revealed no significant effect of physical attractiveness (*F*_(1,25)_ = 0.46; *p* > 0.05), nor the effect of the sex of participants (*F*_(1,25)_ = 0.02; *p* > 0.05). However the main effect of the actor’s sex was significant (*F*_(1,25)_ = 27.7; *p* < 0.001; η^2^ = 0.53), and greater compassion was expressed toward the women than toward the men. Moreover, an interaction between the actor’s sex and attractiveness factors (*F*_(1,25)_ = 5.97; *p* = 0.02; η^2^ = 0.19) was found. Between the female actresses, greater compassion was expressed toward the less attractive actress (*p* = 0.04). No such difference was observed for the male actors (see Figure 3).

The analysis of the RTs revealed a main effect of attractiveness (*F*_(1,25)_ = 25.9, *p* < 0.001; η^2^ = 0.51), only. The subjects responded significantly faster when they declared compassion toward the more attractive actors (*M* = 2.08, SD = 0.09) compared with the less attractive actors (*M* = 2.26, SD = 0.1).

### Neuroimaging Results

**Figure 4:**
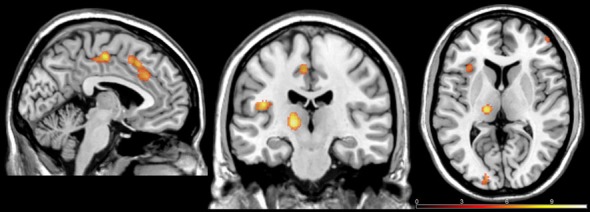
**Brain regions exhibiting activation in the Pain > No Pain at *p* < 0.05 family-wise error corrected (FWE)-corrected**.

Based on previous studies showing brain areas involved in empathy, we performed a whole-brain analysis of the Pain (all of the intensities) vs. No Pain conditions. *T*-test contrast-based analysis revealed increased activity in several brain regions, including the left thalamus, left AI (BA13), left ACC (BA32), right posterior cingulate cortex (PCC; BA31; BA23), bilateral supplementary motor area (SMA; BA6), right cerebellum, right DLPFC (BA46) and right FPC (BA10), caudate nucleus, putamen, and substantia nigra (see Table 1; Figure 4). Furthermore two clusters were activated in brain regions previously shown to be involved in evaluation of attractiveness: left lingual gyrus (BA17) and middle occipital gyrus (MOG; BA18). We did not observed any significant interaction or gender differences in the Pain vs. No Pain contrast.

To address the question of whether attractiveness is a modulator of the empathic brain response we examined the contrast higher attractiveness vs. lower attractiveness within the Pain condition. No significant activation was found when highly attractive models were compared to less attractive ones within the Pain condition. However, we observed a significant interaction between Attractiveness and the Sex of the Actors. The analysis revealed increased activity in the structures which are linking to empathy, including the left thalamus, left AI (BA13), left SMA (BA6), left cerebellum, left DLPFC (BA46), claustrum, caudate nucleus, PG (BA4), bilateral superior temporal gyrus (STG; BA22), supramarginal gyrus (SMG; BA40), middle frontal gyrus (MFG; BA10), left inferior frontal gyrus (IFG; BA9). Moreover, there was also increased activity in left MOG (BA19) and bilateral middle temporal gyrus (MTG; BA39), structures related to evaluation of attractiveness (see Table 2). For all these structures there was greater activity for the less attractive man than for the more attractive man and greater activity for the more attractive woman than for the less attractive woman.

**Table 2:** **Brain regions showing activation Attractiveness × Sex of the Actors at *p* < 0.05 FWE corrected**.

				MNI coordinates		
	Brain region	BA	peak *T*	*x*	*y*	*z*	Cluster size
L	Middle temporal gyrus	BA39	97.2	−36	−74	14	40467
R	Middle temporal gyrus	*	89.3	53	−66	3	
R	Middle occipital gyrus	BA 19	87.4	44	−75	14	
L	Supplementary motor area	BA6	56.1	−29	−6	63	770
L	Precentral gyrus	BA4	40.3	−41	−6	61	
L	Supplementary motor area	BA6	38.2	−26	−10	53	
L	Thalamus	*	49.1	0	−14	17	334
L	Thalamus	*	35.7	−6	−20	15	
L	Caudate	Caudate body	31.2	−12	−4	18	
L	Dorsolateral prefrontal cortex	BA46	46.0	−50	47	10	71
L	Thalamus	*	44.5	−18	−35	9	102
L	Parahippocampal gyrus	BA30	32.5	−26	−38	6	
L	Caudate	Caudate body	42.9	−20	5	27	169
L	Caudate	Caudate body	32.8	−20	14	19	
L	Caudate	Caudate body	42.4	36	−36	35	44
L	Superior temporal gyrus	BA22	40.6	−53	8	3	156
R	Superior temporal gyrus	BA22	38.9	60	9	−12	36
L	Culmen, cerebellum	*	35.9	−8	−29	−21	10
L	Inferior frontal gyrus	BA9	34.6	−42	11	25	164
L	Inferior frontal gyrus	BA9	29.4	−50	12	33	
L	Supplementary motor area	BA6	28.8	−33	11	27	
L	Anterior insula	BA13	34.4	−42	−15	−5	74
L	Claustrum	*	31.6	−36	−4	0	
L	Claustrum	*	28.3	−35	−14	1	
L	Supramarginal gyrus	BA40	33.3	−56	−47	33	22
L	Suplementary motor area	BA6	33.0	−38	−3	31	27
R	Thalamus	Pulvinar	32.7	17	−24	18	17
L	Thalamus	Pulvinar	32.3	−24	−24	6	10
L	Frontopolar cortex	BA10	32.0	−41	59	−5	11
R	Superior temporal gyrus	BA42	31.9	62	−26	17	26

*Abbreviation: L, left hemisphere; R, right hemisphere; BA, Brodman Area. All of the brain regions listed in this table were cluster-corrected and met the significance threshold of p < 0.05. The cluster size is the number of voxels activated in the regional cluster. Only the main peaks of activation within each cluster and their corresponding brain structures are reported*.

### Region of Interest Analyses

Based on previous neuroimaging findings (Lamm et al., 2011; Gu et al., 2013), we conducted additional ROI analyses to examine the interaction between the studied conditions, that is attractiveness and actor sex in the following structures: thalamus, AI, ACC, caudate, and putamen. Just as in the case of behavioral results, a repeated measures ANOVA was used with the following within-subject factors: physical trait (higher attractiveness vs. lower attractiveness) and the actor’s sex (woman vs. man). Once again, for interaction effects the Sidak correction for multiple comparison was computed (*p*-values reported are two-tailed).

There were no main effects of Attractiveness or Actors’ Sex reported in any of the analyzed structures. However, a number of interaction effects were revealed. All of the significant interaction effects are described below.

**Figure 5:**
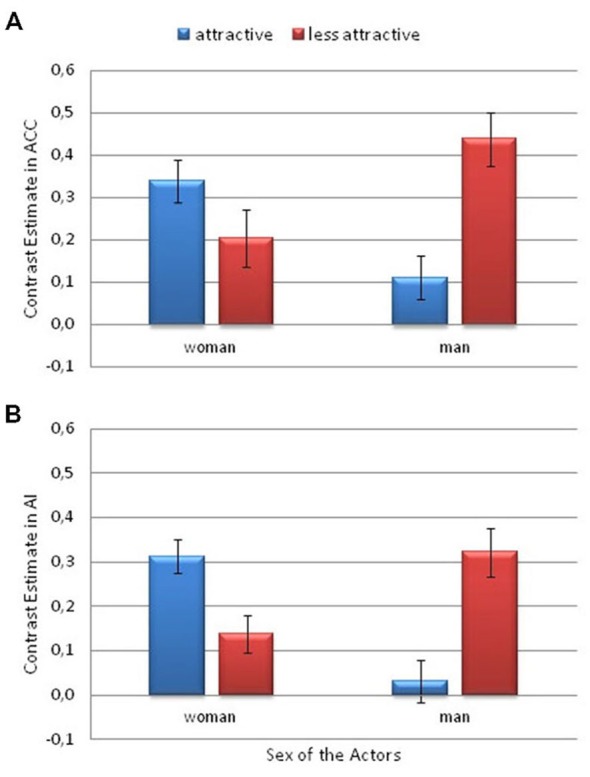
**Contrast estimates depicting the significant attractiveness and actor sex interaction**. This is from the Pain > No Pain contrast. **(A)** in the anterior cingulate cortex (ACC) and **(B)** the anterior insula (AI). Error bars represent the standard error of the mean.

The ROI analysis of the signal intensity revealed significant Attractiveness × Actor Sex interactions in the ACC (*F*_(1,26)_ = 5.45; *p* = 0.028; η^2^ = 0.17) and AI (*F*_(1,26)_ = 5.90; *p* = 0.022; η^2^ = 0.19; see Figure 5). There was greater activity for the less attractive man than for the more attractive man in the ACC (*p* = 0.016) and AI (*p* = 0.048). Among the more attractive actors, greater activity was observed for the woman than for the man in the ACC (*p* = 0.06) and in AI (*p* = 0.015).

**Figure 6:**
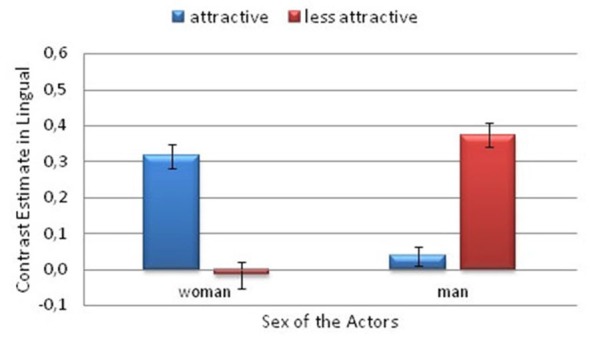
**Contrast estimates depicting the significant attractiveness and actor sex interaction**. This is from the Pain > No Pain contrast. In the lingual gyrus. Error bars represent the standard error of the mean.

A significant interaction between Attractiveness and Actor Sex was also observed in the lingual gyrus (*F*_(1,26)_ = 24.9; *p* < 0.001; η^2^ = 0.49). There was greater activity in the lingual gyrus for the less attractive male actor than for the more attractive male (*p* = 0.001). However, the activity was greater for the more attractive woman than for the less attractive woman (*p* = 0.005). Among the more attractive actors, greater activity was observed for the woman than for the man (*p* = 0.003). The opposite pattern was observed among the less attractive actors, that is, greater activity was observed for the man than the woman (*p* < 0.001; Figure 6).

**Figure 7:**
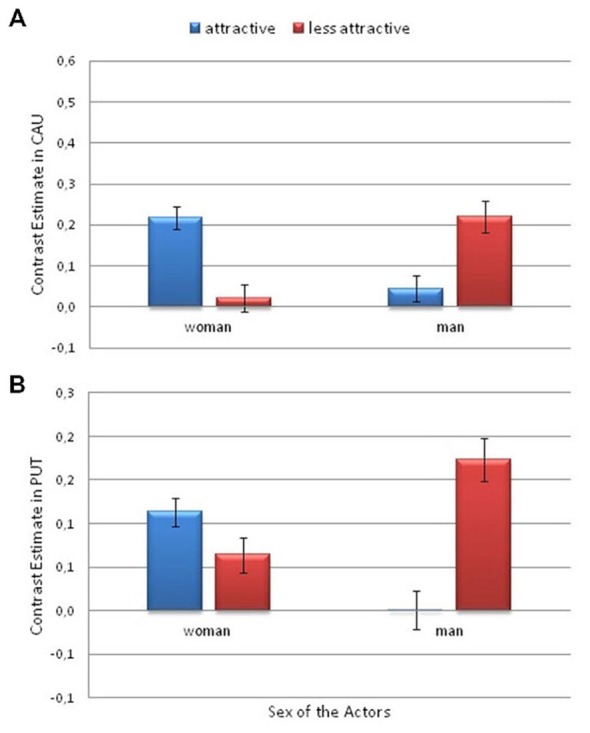
**Contrast estimates depicting the significant effects of attractiveness and actor sex**. This is from the Pain > No Pain contrast. **(A)** in the caudate nucleus and **(B)** in the putamen. Error bars represent the standard error of the mean.

Additionally, a significant interaction between Attractiveness and Actor’s Sex was found in the caudate nucleus (*F*_(1,26)_ = 7.33; *p* = 0.012; η^2^ = 0.22). There was greater activity for the more attractive female actor than for the less attractive female (*p* = 0.031). However, the activity was greater for the less attractive man than for the more attractive man (*p* = 0.042; Figure 7A).

Finally, a significant interaction between Attractiveness and Actor’s Sex was observed in the putamen (*F*_(1,26)_ = 6.6; *p* < 0.017; η^2^ = 0.2). There was greater activity for the less attractive male actor than for the more attractive male (*p* = 0.011; Figure 7B).

## Discussion

To the best of our knowledge, the present study is the first attempt to examine the influence of attractiveness on the level of empathy using fMRI methods. The first of our hypotheses was confirmed. There is an activation in the brain area related to empathy for pain when watching more attractive and less attractive models showing pain responses on their faces. We observed increased blood oxygenation level-dependent (BOLD) signals in the brain structures AI and ACC. Our findings support the well-documented evidence that these brain regions are essential for empathetic processing (Singer et al., 2004; Fan et al., 2011; Lamm et al., 2011). These regions play an important role in the emotional and physical distress that accompanies painful stimulation (Singer et al., 2004). We observed activation in the FPC and DLPFC, supporting the assumption that, during empathizing with pain shown on the faces of observed models, the perspective-taking process and understanding the emotions of other people take place (Schulte-Rüther et al., 2007; Decety and Meyer, 2008; Bernhardt and Singer, 2012; Klimecki et al., 2013). They play roles in imagining other people’s feelings (Schulte-Rüther et al., 2007; Light et al., 2009). Moreover, we observed increased activation in the striatum, which is the putamen and caudate nuclei, during observation of the models expressing pain responses. These structures are connected to several other regions, including the ACC and prefrontal control regions (Haber and Knutson, 2010), and constitute the so-called dopamine-mediated brain circuit that is involved in motivational processing (Haber and Knutson, 2010). Anticipatory striatal activity is thought to reflect motivational salience and has been linked to the motivation for both reward and punishment avoidance (Schultz et al., 1992; Salamone, 1994; Cremers et al., 2015). Further, the activity of the dorsal striatum may be associated with the processing of compassionate emotional meanings (Roy et al., 2012). Adopting compassionate attitudes toward pictures of sad faces augments activations in the ventral striatum and the VTA/SN (Kim et al., 2009; Klimecki et al., 2012, 2013). Additionally, activations in the middle insula, dorsal ACC and the striatum (comprised of the putamen, globus pallidus and caudate nuclei) have been observed in romantic and maternal love (Bartels and Zeki, 2000, 2004; Aron et al., 2005; Beauregard et al., 2009) and affiliation (Vrticka et al., 2008; Strathearn et al., 2009). These results have been interpreted as reflecting the rewarding nature of experiencing love and warmth, that is, caring even when faced with the suffering of others. However, it is also plausible that the reward responses are just increased pride or self-congratulatory responses from feeling good about being compassionate. To decide which of the two interpretations is correct requires further research. Furthermore, the activations observed in the lingual gyrus and MOG may suggest that the presented visual stimuli were, in fact, of high emotional valence (Vartanian and Goel, 2004). This finding is in line with that of several other studies that have highlighted the roles of the primary and associative visual cortices in the processing of pictures (Paradiso et al., 1999) and faces (Iidaka et al., 2002) varying in emotional valence. Activation in MOG was correlated with the attractiveness rating for faces, which was attributed to processing faces that vary in rewarding properties (Vartanian et al., 2013).

The second hypothesis did not receive support in our data. Regarding differences in the perceptions of a person as attractive or unattractive, we did not observe any significant differences in brain activation at the level of the whole brain and the ROI analysis. However, the signal intensities in the structures (i.e., the AI, putamen and caudate) were significantly affected by the interaction of the attractiveness and sex factors, instead. Greater activity in the structure linking with empathic brain and evaluation attractiveness was observed for the less attractive man than for the more attractive man, and for the attractive woman compared with the attractive man. This is an intriguing finding that could suggest that being an attractive man reduces ones likelihood of receiving empathy. Attractive male faces are typically related to enhanced masculine facial characteristics (Brown et al., 1986; Cunningham et al., 1990; Grammer and Thornhill, 1994; Dunkle and Francis, 1996; Johnston et al., 2001; Penton-Voak et al., 2001; Rennels et al., 2008). This masculinity is often perceived as a cue that indicates a high level of dominance, unsuitability as a partner (Boothroyd et al., 2007), and characteristics such as coldness and minimal cooperativeness (Perrett et al., 1998). Similarly, Johnston et al. (2001) found that increasing masculinity increases perceptions of antisocial traits, which may be relevant to relationships and personal investment (Perrett et al., 1998). Consequently, less attractive, less masculine men might evoke more empathy than more attractive men. Indeed, our less attractive male model was judged to be less masculine than our attractive male model. Regarding the women, a different effect might have occurred. Femininity contributes to female attractiveness (Thornhill and Gangestad, 1993; Grammer and Thornhill, 1994; Fink and Penton-Voak, 2002; Little et al., 2002; Rhodes, 2006). Auster and Ohm (2000) reported that femininity is associated with adjectives such as gentleness, sensitivity, shyness, tenderness and warmth. Thus femininity might have evoked greater empathy in the participants, which would explain the greater activations in the structures that are regarded as parts of the empathic core, that is the ACC and AI (Fan et al., 2011; Lamm et al., 2011), in the case of the more attractive woman, who was judged as more feminine than the less attractive female model. Similar results were obtained in the striatum, which is involved in both compassion and in the brain reward circuit. The activity in the caudate nucleus and putamen were greater for the less attractive man than for the more attractive man and for the more attractive woman compared with the more attractive man. This pattern may represent increased positive appraisals of the aversive stimuli of the less attractive man (Wager et al., 2008) due to enhancements of the reward value of the victim’s well-being (i.e., caring; Weng et al., 2013). It would be interesting to determine how modifying attractive faces in terms of more male or more female characteristics would affect the assessment of attractiveness and the level of empathy.

The third hypothesis, concerning the effect of attractiveness of female model on the level of empathy in men did not receive support in our research. We did not observe any sex differences in relation to the presentation of the models representing an opposite sex, neither at the behavioral level nor at the level of fMRI analysis. The research conducted so far has revealed the different brain activation in men and women in response to attractive models. However, the task performed during fMRI scanning was different, and consisted in evaluation of the level of attractiveness of the models (O’Doherty et al., 2003; Winston et al., 2007; Vartanian et al., 2013). In men, there was an activation in brain structures which are considered a part of the reward system: NAcc and mOFC. We did not observe activation of these structures in our research. It is possible that observing unpleasant emotions, such as pain, even in attractive models such as women, could reduce the effect of attractiveness. For example, it was revealed that negative emotional expressions, such as sadness, tend to reduce high levels of attractiveness (O’Doherty et al., 2003). Evolutionary psychology studies suggest that beauty is valued more highly in females than males (Buss, 1994/2003), which might lead observers to empathize more strongly with the attractive woman than the man. When confronted with signs of pain however, the beauty of women may be less important evolutionally, because the condition of survival may be more important than reproductive success.

Regarding behavioral performance, we observed differences in the levels of declared compassion (empathic concern) toward the models expressing pain responses that depended on the sex and the attractiveness level of the model. More compassion (empathic concern) was declared toward the less attractive woman than the more attractive woman. There was no difference in the levels of declared empathy toward the more attractive and less attractive man. Additionally, we found that the time required to assess the level of compassion toward the less attractive actors was longer than that required for the more attractive actors. The result may suggest that the participants thought more when assessing their compassion toward the less attractive models.

Notably, the results obtained in the behavioral portion of this study seem to contrast with the results from the fMRI portion of the study. First, more self-rated compassion was declared toward the women than toward the men. Secondly, more compassion was declared toward the less attractive woman than toward the less attractive man, whereas the brain activity exhibited the opposite pattern. This disparity may result from the discrepancy between the effect of social bias on reflexive inferences about our own compassion level vs. the automatic responses of the empathic brain. This self-reflective behavior might be motivated by the need to fulfill specific social expectations, such as being regarded as a person who has lots of empathetic concern toward people, especially towards those who, based on social judgments, require more help and support. Such a reflexive behavior can be regarded as a part of an explicit attitude that is driven by gender stereotypes in which femininity is associated with sensitivity, tenderness and so on, and evokes a stronger protective tendency (Auster and Ohm, 2000; Prentice and Carranza, 2002). However, one should keep in mind, that the results of self-rated compassion level are based on the participants answer to a single question. On the other hand, at the level of the fMRI analysis, the process of empathizing was not controlled by social stereotypes or by the tendency to present oneself as a good and righteous person, because stronger activations were observed in the automatic, consciously uncontrolled action of the empathic brain in response to the more attractive woman and the less attractive man.

In conclusion, this study showed, for the first time, that the attractiveness and sex of a model constitute an important modulator of pain empathy. The activity of brain structures related to empathizing were higher when the participants watched the less attractive man and more attractive woman than when they watched the more attractive man and the less attractive woman. Understanding of yet another factor related to the level of empathy for pain brings us closer to elaborating better psychotherapeutic methods or social persuasion techniques which may increase the level of empathy in society. Rising empathy levels may result in more altruistic and prosocial behaviors, which are crucial for the survival of our society.

## Conflict of Interest Statement

The authors declare that the research was conducted in the absence of any commercial or financial relationships that could be construed as a potential conflict of interest.
